# A comparative study of cranial, blunt trauma fractures as seen at medicolegal autopsy and by Computed Tomography

**DOI:** 10.1186/1471-2342-9-18

**Published:** 2009-10-16

**Authors:** Christina Jacobsen, Birthe H Bech, Niels Lynnerup

**Affiliations:** 1 Section of Forensic Pathology, Department of Forensic Medicine, University of Copenhagen, Frederik V Vej 11, DK-2100 Copenhagen Ø, Denmark; 2 Diagnostic Centre, Department of Radiology, Copenhagen University Hospital, Blegdamsvej 9, DK-2100 Copenhagen Ø, Denmark

## Abstract

**Background:**

Computed Tomography (CT) has become a widely used supplement to medico legal autopsies at several forensic institutes. Amongst other things, it has proven to be very valuable in visualising fractures of the cranium. Also CT scan data are being used to create head models for biomechanical trauma analysis by Finite Element Analysis. If CT scan data are to be used for creating individual head models for retrograde trauma analysis in the future we need to ascertain how well cranial fractures are captured by CT scan. The purpose of this study was to compare the diagnostic agreement between CT and autopsy regarding cranial fractures and especially the precision with which cranial fractures are recorded.

**Methods:**

The autopsy fracture diagnosis was compared to the diagnosis of two CT readings (reconstructed with Multiplanar and Maximum Intensity Projection reconstructions) by registering the fractures on schematic drawings. The extent of the fractures was quantified by merging 3-dimensional datasets from both the autopsy as input by 3D digitizer tracing and CT scan.

**Results:**

The results showed a good diagnostic agreement regarding fractures localised in the posterior fossa, while the fracture diagnosis in the medial and anterior fossa was difficult at the first CT scan reading. The fracture diagnosis improved during the second CT scan reading. Thus using two different CT reconstructions improved diagnosis in the medial fossa and at the impact points in the cranial vault. However, fracture diagnosis in the anterior and medial fossa and of hairline fractures in general still remained difficult.

**Conclusion:**

The study showed that the forensically important fracture systems to a large extent were diagnosed on CT images using Multiplanar and Maximum Intensity Projection reconstructions. Difficulties remained in the minute diagnosis of hairline fractures. These inconsistencies need to be resolved in order to use CT scan data of victims for individual head modelling and trauma analysis.

## Background

Computed tomography (CT) scanning of bodies prior to medico legal autopsy has become a powerful tool in several forensic institutes throughout the world [1-3]. Indeed, there may be a number of advantages to performing a CT scan before a medico legal autopsy. In forensic pathology as well as in clinical settings the investigation of head trauma is based on a combined analysis of the present external lesions, cranial fractures and intracranial lesions. The scans of the head are suitable to visualize lesions of especially bone, but also some soft tissue and most cerebral pathologic changes or lesions [4]. Fractures, intracranial haemorrhages and hematomas can be demonstrated either 2- and 3-dimensionally, providing a general overview of simple as well as complex lesions, e.g., gun-shot lesions or large fracture systems [3,5-8]. However, especially minor lesions of the soft tissue, bone or intracranial content can be difficult to diagnose on CT scan which has to be kept in mind when investigating head trauma and the corresponding injury mechanisms.

Attempts have been made to use the acquired CT data for detecting causal relationships either by illustrating and interpreting lesions based on CT images [6,8] and other tools [9], or by using the data to attempt biomechanical analysis [10] to analyze lesions. Due to the nature of the technique, CT scanning also allows for the collection of spatial data of the cranium, which potentially could be used in the development of new analytical tools in forensic pathology and injury biomechanics [11,12]. Biomechanical models of the head are increasingly used in the forensic sciences to analyze injury mechanisms [13-15]. CT scan data may be particularly useful for creating biomechanical models [16,17] of body parts, e.g. the cranium [18-21], because they may provide precise spatial, digital data which represent the complex anatomic structures of the cranium of the single individual. The possibility of being able to develop either standard or individual cranial models subject to retrograde trauma analysis could open up for new prospects regarding medico legal case work both in adults and children.

We have shown that it may be difficult to visualize non-dislocated fractures of the cranium on CT scans performed in a routine setting [22], especially when the fractures are located in the cranial base. Forensically important information about the whole fracture system and possible impact points may then be lost in the cases in which these fractures provide clues about the causative forces. If CT scan data are to be used in future retrograde biomechanical modelling, we need to not just examine the overall diagnostic agreement between CT scanning versus autopsy, but also to examine in more detail exactly how fractures seen on CT scans match autopsy finds.

To this end we performed a detailed analysis of 14 cases with limited cranial fractures by carefully recording the fractures as diagnosed on CT scans post processed with Multiplanar reconstruction (MPR) [23] and Maximum Intensity Projection (MIP) [24], as well as by detailed recording of the fractures at autopsy (drawing and photos). We further tried to quantify the differences by tracing the fractures as seen at autopsy using a 3-dimensional digitizer and merging this 3-dimensional data set with a 3-dimensional reconstruction of fractures diagnosed on CT scan.

## Methods

The study included fourteen cases (13 male, 1 female; age-range: 19-82 years, mean 47 years) with neurocranial fractures with a limited extension, caused by blunt violence. Nearly all of the cases were accidents, while one was the result of an assault with a baseball bat (see table 1). In one case the manner of death was unknown.

**Table 1:** The different trauma mechanisms related to the case numbers.

Trauma mechanism	Case no.
Assault	#2
Fall -- ground level	#10
Fall -- 2-3 metres	#8, #11, #12, #13
Fall - >4 metres	#14
Fall -- unknown height	#1, #9
Traffic -- hit	#3, #5, #7
Traffic -- hit and fall	#4, #6

### The CT scan and the autopsy

Each body was scanned using an MSCT-scanner (Siemens Somatom Plus 4 Volume Zoom) prior to the autopsy. The scan was obtained in an axial plane using a slice collimation of 4 × 1 mm, pitch 0.65, 120 KV, mAs ~150 and bone algorithm (H60s).

At the autopsy, the cranium and the fractures were photographed and registered on a schematic drawing. Also, the fracture characteristics, extension and anatomic location were registered. For comparative purposes the neurocranium was divided into the following major anatomic regions: the vault (squamous part of the frontal bone, the parietal bones, and the squamous part of the temporal bone) and the cranial base. The cranial base was further subdivided into the posterior fossa (occipital bone), the medial fossa (petrous part of the temporal bone, greater wing of the sphenoid, sella turcica) and the anterior fossa (orbital part of the frontal bone and the lesser wings of the sphenoid).

### The CT readings

Two CT readings were performed. The first diagnostic fracture reading was performed by a forensic pathologist (CJ) on the Siemens scanner workstation prior to the autopsy. The axial images and a Multiplanar reconstruction (MPR) of the sagittal and coronal image planes with reconstruction increment of 0.5 mm were used. The second diagnostic reading of the same CT images was performed in cooperation with a board certified radiologist (BLH) on an Agfa Impax DS 3000 workstation in consideration of autopsy findings. In addition to the MPR, a thick (5 mm) MIP was performed, and in selected cases a curved MPR. Due to technical difficulties case #13 did not undergo a second evaluation. Discontinuity or dislocation of the bone was defined as being a fracture. In some cases there were suture diastases and to avoid interpreting these as fractures the width of the suture was compared to the parallel sutures. Intracranial air or blood in the sinuses served as an indicator for possible fractures, but if a discontinuity of the bone was not visible the likely associated fracture was not registered.

### The comparison between the CT readings and the autopsy results

In order to compare the extension and anatomic localisation of the fractures, the fracture diagnosis of the first and second CT scan evaluation were registered on the schematic drawings from the autopsy. It was also noted whether the fractures were uni- or bilateral and, on the CT scans, whether there was fluid in the sinuses or mastoids. This clearly showed whether congruence between the autopsy and the reconstructed CT images (MPR, MIP and curved MPR) existed. The data were analyzed regarding the recognition of an overall fracture system, providing important information about the traumatology in forensic casework, and whether the overall as well as the minute fracture diagnosis was correct compared to the autopsy results.

**Figure 1:**
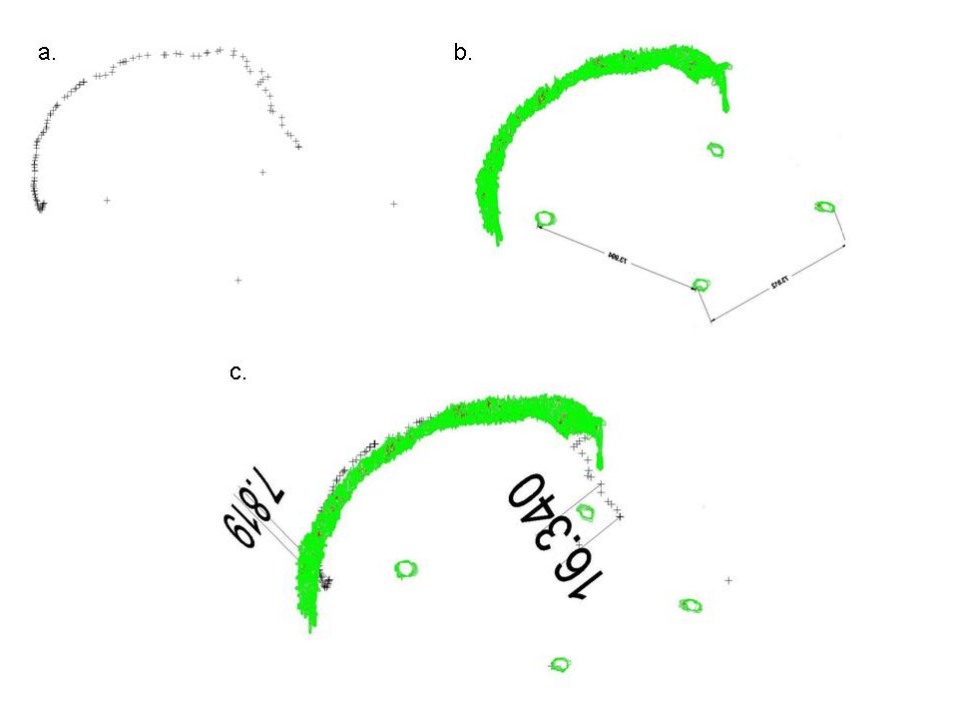
**a, b and c. The 3-dimensional data set from case #6 with the digitised fracture at the autopsy (a), the segmented fracture from the CT image (b) and the merged two data sets (c)**. The dimensions are in mm and represent only some of the measurements. The 2-dimensional representation of the 3-dimensional image causes distortion.

In order to further quantify location and congruence between autopsy and CT scan we selected five cases, and their 3-dimensional fracture registrations based on the first reading of the CT scan data and on the autopsy were merged. We did this by performing a 3-dimensional fracture registration at the autopsy by tracing the fractures using a digitiser (Patriot^® ^Polhemus, US). The CT scan data were transferred to Mimics^®^, a software programme which allows single slice editing and segmentation, enabling us to segment the fractures as seen on the CT images. The two obtained data sets, from the autopsy and the CT scan, were transferred to Design CAD 3D Max 15^®^, a computer aided design package, and merged (see figure 1a, b and 1c).

### The material

At the autopsy the majority of the cases (12/14) comprised linear cranial fractures. There was also a case with a depressed fracture in the vertex and one case with a local comminute fracture in the occiput. In half the cases several separate fractures were present per case, e.g., a linear fracture involving the posterior fossa and a separate fracture involving the medial fossa. Also more than half (n = 9) of the fractures comprised a varying number of ramifications (n = 1-5), which at the autopsy were seen as undislocated hairline fractures. Only a few of all the fractures were dislocated (n = 4) and the fracture width varied from 4 mm - 1 mm. Suture diastases was involved in 5 cases and involved the lambdoid, squamous and sphenofrontal suture.

The Study was approved by the Ethics Committee for Copenhagen and Frederiksberg, Denmark KF01-154/04.

## Results

### The anatomic localisation of the cranial fractures as diagnosed at the autopsy

It was seen at the autopsy that the basal fossae and the vault were affected in 34 instances (see table 2). The anterior and medial fossae were affected bilaterally in half the cases, while bilateral fractures in the posterior fossa only occurred once. Fractures involving the anterior fossa bilaterally (cases #6, #7, #13) never crossed the midline, and in two cases (cases #3, #12) only involved the orbital loft of the frontal bone. The unilateral fractures in the anterior fossa (cases #1, #2, #11) were, except from one (case #11), continuations of fractures from the medial fossae (cases #1, #2) and were located in the sphenoid. Fractures involving the medial fossae (cases #1-7, #10-13) were separate fractures in two cases (cases #10, #13) but were mostly continuations of fractures from the posterior fossa (cases #5, #6, #11) or the theca (cases #1-4, #7). Four of the bilateral fractures in the medial fossae traversed the sella turcica (cases #3-5, #12).

**Table 2:** Case based number of fractures in the anatomic entities of the neurocranium

Fractures	Base Anterior fossa	Base Medial fossa	Base Posterior fossa	Vault	Total
Unilateral	4	5	7	5	21
Bilateral	3	6	1	3	13

Total	7	11	8	8	34

### The anatomic localisation of the cranial fractures as diagnosed at the CT readings

**Figure 2:**
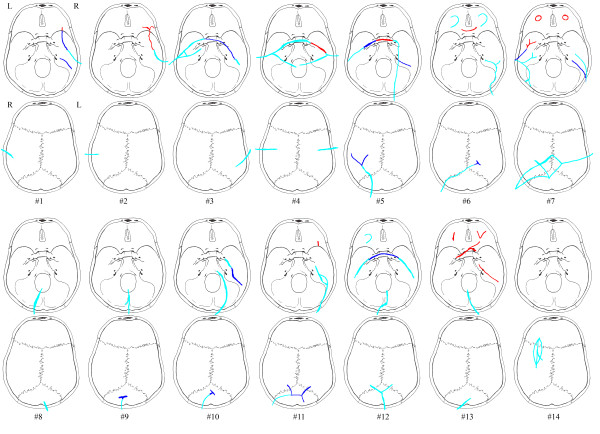
**Registration of the case based fracture congruity between autopsy and CT**. The fractures diagnosed at autopsy are represented by all colours (red, light blue and blue). The light blue colour by itself represents the part of the fractures diagnosed during the first reading; the dark blue colour represents the additional part of the fractures diagnosed during the second reading; the red colour represents the part of the fractures which were not diagnosed at all at the CT readings.

The anatomic localisation and extent of the fractures was diagnosed completely during the first reading of the MPR CT images in two of the fourteen cases (cases #8, #14) (see figure 2). Case #8 comprised a simple linear fracture in the occipital bone oriented anterior-posterior. The fracture in case #14 was depressed, oriented anterior-posterior and located in the vertex. In ten cases (#1-6, #9-12) there was a partial fracture diagnosis based on the MPR CT images, but the overall fracture system was recognized.

By performing the second reading of the MPR and MIP CT images of 13 of the 14 cases (cases #1-12, #14) the fractures of the cases #1, #3, #10 and #12 were diagnosed completely. In one case (case #7) important information, regarding contre-coup fractures, was missed on the CT-scan since fractures of the eye loft in the anterior fossae were not diagnosed.

### The quantification of the fracture extent based on the autopsy findings and the CT scan readings

Table 3 shows the quantification of the fractures extent as measured by digitiser at the autopsy compared to the measurements performed on the CT scans after the first reading. The table shows that up to 50% of the full extent of the fractures was missed in one case (case 1). The missed fractures were hairline fractures. In cases #9, #6 and #11 the Y-, T- and H-shaped hairline fracture of the impact point was missed. In cases #11, #6 and #1 the hairline fractures located in the medial fossa, both in the petrous bone and the sphenoid, were not diagnosed. Case #14 represented the only depressed fracture in the material and in that case the diagnosis was correct.

**Table 3:** Comparison of the digitised fracture length to the fracture length as measured on CT images of the first reading

**Case no**.	Digitiser	CT (cm)	Digitiser - CT subtraction sum (cm)	Anatomic localisation of miss
#9	13.4	8.3	5.1	Occipital bone T-shaped hairline fracture
#11	22.2	17.5	4.7	Petrous bone, minor wing of the sphenoid (~1 cm each) and H-shaped fracture in the occiput
#6	20.3	18.1	2.2	Parietal bone (0.6 cm) and petrous bone (1.6 cm)
#1	22.1	10.3	11.8	Petrous bone ~3 cm and the great sphenoid wing (~8.8 cm)
#14	6.7 (length) 2.4 (width)	5.8 (length) 1.7 (width)	0.9 (length) 0.7 (width)	Depression fracture of the parietal bone

The fracture of the eye loft in case no. 6 was not digitised

### The analysis of CT scan based fracture diagnosis by using different reconstructions

**Figure 3:**
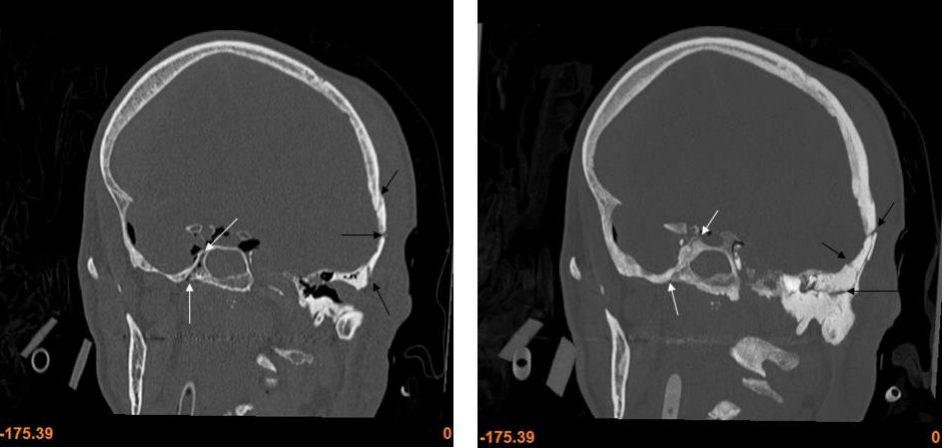
**MPR (right) and MIP (left) coronal images of fractures in the left temporal bone and right great wing of the sphenoid**. The MIP provides a very good overview of the fractures extension. The fractures in the temporal bone are indicated by black arrows and the fractures in the great wing of the sphenoid are indicated by white arrows.

**Figure 4:**
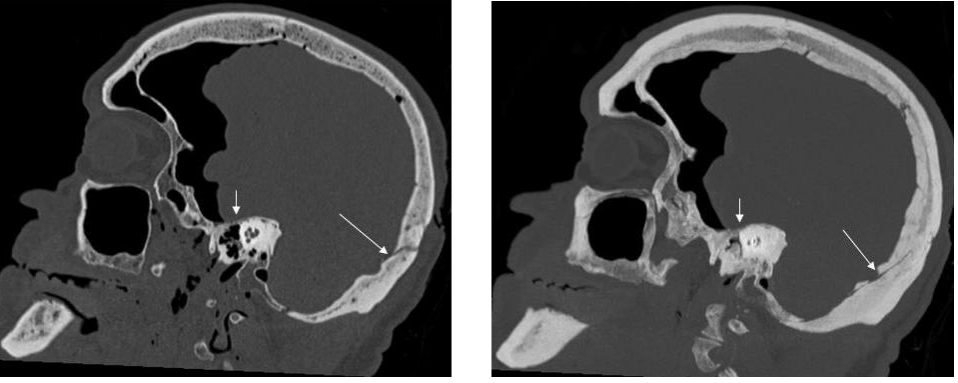
**MPR (right) and MIP (left) sagittal images of fractures in the occipital bone and petrous part of the temporal bone**. The MIP provides an overview of the fractures extension and the hairline fracture in the petrous (short white arrow) and occipital bone (long white arrow).

The missed fractures on the MPR CT images were characterised by being undislocated hairline fractures or ramifications of the wider fractures. Not surprisingly most of the missed fractures were located in the basal medial and anterior fossae (see table 4). This is exemplified in the cases #2, #4-7 and #13 (see figure 2), of which cases #7 and #13 are with severe incongruity in fracture diagnosis. Using thick MIP reconstructed CT images at the second evaluation did not facilitate the diagnosis of fractures in the minor wings of the sphenoid nor in the pars orbitalis of the frontal bone. The greatest advantage of using thick MIP reconstructed CT images was achieved in the medial fossa (see figure 3) and in the vault when visualising ramifications related to impact points. In the medial fossa the usage of thick MIP improved the diagnostic frequency of fractures in the petrous part of the temporal bone (see figure 4) and the greater wings of the sphenoid by approximately 50%. The main fractures in the basal posterior fossa and the vault, except from five ramifications (cases #5, #6, #9-11), were diagnosed in the first evaluation. The ramifications were associated to impact points and were therefore important in the forensic casework. During the second evaluation, the usage of curved MPR on the CT images made the diagnosis of two of the ramifications possible (case #5 and #11) while the remaining ramifications were diagnosed by using thick MIP (case #6, #9 and #10).

**Table 4:** Number of fractures in each bone as diagnosed by autopsy versus CT-scan

Region	Anterior fossa		Medial fossa			Posterior fossa	Vault			Total
**Bone**	**Frontal**	**Sphenoid**	**Temporal**	**Sphenoid**	**Sphenoid**	**Occipital**	**Frontal**	**Parietal**	**Temporal**	
	**Pars orbitalis**	**Minor wings**	**Petrous**	**Greater wings**	**Sella turcica**				**Pars squamosa**	
	
Autopsy	7	5	15	15	4	8	1	12	9	76
1^st ^CT reading	3	0	6	5	2	8	1	9	8	42
2^nd ^CT reading	3^a^	0	11	11	3	8	1	12	9	58

^a^One case with two fractures of the eye loft did not undergo the second evaluation

### The analysis of secondary signs of fracture

In several cases there were indications of fracture on the CT images with fluid in the mastoid cells (case #10, #4, #1, #12, #13), which at the autopsy always was associated with fracture in the same petrous bone. In four of these cases there was also fluid in the sphenoid sinus on the CT images (case #4, #12, #13) and/or the ethmoid sinus (case #4, #1, #12). In two cases fracture of the sinus walls was diagnosed on the CT image. In only one of these cases an associated fracture in the anterior fossa was not diagnosed at the autopsy (case #4).

## Discussion

Our study showed that the forensically important fracture systems to a large extent were diagnosed on CT images using MPR and MIP reconstructions. In this technical study we focused only on minor fracture systems which limited the number of included cases and restricted the usage of statistical methods including the evaluation of diagnostic sensitivity/specificity. The autopsy findings were known during the second CT scan reading which made a problem based reading with the radiologist possible identifying and targeting difficult diagnostic areas. Future larger blinded diagnostic studies could evaluate the congruence between autopsy and CT scan images further. Also the usage of new generation CT scanners with the technical ability to produce isotropic data would probably improve the diagnostic accuracy [25,26].

The CT scan based recognition of fractures located in the basal cranial fossa and also the cranial vault is important both in the clinical setting regarding treatment efficiency [27-29] and in medico legal material to be able to analyze injury mechanisms. However, in clinical settings the diagnosis of hairline fractures is mostly not essential as long as there are no clinical symptoms or complications [30]. The medico legal material in this study reflected the trauma severity with a fracture involvement of the medial basal fossa in 80% of the cases, which in this study was an area in which fracture diagnosis was difficult. Often both the pars petrosa of the temporal bone and the sphenoid bone were affected simultaneously either unilaterally or bilaterally and there were also a few cases with transphenoidal fractures resulting in involvement of both fossa, which in clinical studies is interpreted as the result of severe head injury [31-35]. Unger et al. [28] found in a clinical study that fractures of the cranial base predominantly were located in the sphenoid bone and to some extent in the temporal bone. In this study most fractures were identified in the greater wings of the sphenoid involving the orbital surface while fractures of the cerebral and temporal surfaces were less common. This finding might be related to the difficulties exemplified in this study in visualising these fractures on CT images. In our study none of the isolated fractures of the minor wing were diagnosed, which is in concordance with the study of Unger et al [28] in which only a few fractures of the minor wings of the sphenoid were diagnosed.

With the combination of MIP, MPR and in some instances curved MPR the identification of fractures at possible impact points was deemed acceptable. It is known that widths of linear fractures at impact sites can be narrower than at locations further away from impact in the same case [18]. The recognition of a possible characteristic fracture corresponding to the impact point in the cranium and the correlation to possible scalp lesions and instruments is important for the casework, both from a biomechanical [18] and a forensic viewpoint.

The results implied difficulties regarding diagnosis of fractures involving the anterior fossa. In all our cases the fractures were confined to either side of the eye loft without midline crossing and were interpreted as being the result of an impact to the back of the head, i.e. contre-coup fractures of the eye loft [36-39]. In most clinical studies fractures of the eye loft are associated with trauma to the facial or frontal region [40-43] producing transverse and longitudinal fractures [44,45] and we are not aware of clinical studies which mention these characteristic fractures after occipital impact. In a forensic routine setting both fracture of the anterior fossa and lesions of the cerebral temporal and frontal lobes in conjunction with occipital impact would be regarded as contre-coup lesions and thereby indicative of this specific injury mechanism. Further studies are needed to elucidate how often cerebral lesions and/or fractures of the eye loft occur in impacts to the back of the head.

The difficulties regarding diagnosis of fractures involving the anterior and medial fossa is also known in the clinical setting [27]. Schuknecht et al [23] stress the use of correct protocols when attempting to diagnose fractures of the bones in the medial and anterior fossa (thin collimation (0.75-1 mm) and 2D MPR with contiguous 2 mm slices in the axial and coronal plane) and especially high resolution (0.5-0.75 mm) for evaluation of the pars petrosa of the temporal bone. Philipp et al [46] found that thin MPR obtained from thin collimation (2 × 0.5 mm) was superior in subtle fracture detection compared to collimation of 4 × 1 mm in midline facial fractures.

Other 3-dimensional reconstructions aside from MIP [24] were not used in this study since the diagnostic improvement by using these studies for visualizing non-dislocated and hairline fractures was not considered to be substantial compared to the 2-dimensional MPR images conf. [40,47,48]. However recent studies have shown a diagnostic improvement for particularly pathological changes in the temporal bones by using Volume Rendering reconstructions [49]. The use of high-resolution MPR's based on 0.625 mm collimations in a problem-based manner has also been found to improve the diagnostic frequency [50].

In this study we also wanted to try to more precisely measure the differences in determining fracture extent based on either CT scanning (MPR) and by direct inspection at autopsy. We were able to do this in five cases, and to our knowledge this represents the first such attempt at direct quantification. Fracture length discrepancies were thus measureable for hairline fractures. We feel that such precise quantification is necessary if CT data is to be used in future forensic, biomechanical injury modelling and Finite Element Analysis of minor fracture systems. One perspective of these techniques is the ability to perform retrograde injury modelling based on the specific case at hand, thereby complementing the general model based approach (see Raul et al. for an overview)[13]. While finite element models in forensics and accident analysis already have been applied to injury simulation [15,51,52], the retrograde analyses will depend much on the correct capture of the full fracture extent and impact area. Capturing less than half of the full fracture extent will necessarily result in a lower calculated impact force, and not capturing the fracture pattern correctly may also result in a wrong interpretation of the causative injury. Clearly there is a need to extend the quantification to a larger sample and other cranial fracture patterns. Further studies of standard models and simulations are also necessary to accumulate data on head injury biomechanics and validate the head models [51].

An indication for a fracture being present involving the basal cranial fossae can be the identification of intra cranial air [53,54], fluid in the sinuses [55] or opacification of the mastoid cells [50]. This was also the case in our material and these pathological changes led to fracture diagnosis in most of these cases. Connor et al. [50] found that the specific use of high-resolution MPR's upon diagnosis of basal cranial fractures or indications hereof (opacified mastoid cells, etc.) on 5 mm axial images led to a higher diagnostic frequency. In our material there was also one case with fluid level in the sphenoid and ethmoid sinuses in conjunction with an occipital impact. There was no associated fracture of the anterior fossa. Fractures of the sinus walls are difficult to diagnose during an autopsy and in these cases CT images are of great advantage. Our material was too small to explore whether fractures of the sinus walls could be associated with impacts in the occiput and how often they occur without associated fracture of the anterior or medial fossa diagnosed during the autopsy. Geserick et al. [38] found in a prospective study that the orbital medial wall, roof and basal wall contained contre-coup fractures relating to occipital impacts. Also other authors have found fractures of the orbital roof in similar cases [36,37]. It remains to be established how often fluid in the mastoids or the sinus is associated to fractures and whether this finding in the sinus alone is as relevant as contre-coup fractures in the eye loft for possibly differentiating between a blow to the head or impact to the moving head (e.g. fall) [36].

Collaboration with a radiologist (BHB) increased the diagnostic frequency of the cranial fractures. During the second reading the difference between a clinical and forensic approach towards diagnosis of cranial fractures was clearly demonstrated. This emphasized the fact that forensic radiology should be an interdisciplinary specialty which will be dependent on input and knowledge from both specialties to evolve further [1,56].

## Conclusion

Our study showed that the forensically important fracture systems to a large extent were diagnosed on CT images using MPR and MIP. The usage of the various reconstructions and the collaboration with a radiologist was beneficial and necessary in this type of cases with non-dislocated fractures and hairline fractures. Difficulties remained in the minute diagnosis of hairline fractures located especially in the anterior or medial fossa. This was exemplified by merging the digitised autopsy data with the data from the CT scan. Using MIP reconstruction, and in selected cases curved MPR, especially focusing on the fossae and at the impact points in the vault or occipital bone, lead to an increase in the diagnostic frequency, which in turn lead to an improvement of the diagnostic possibilities regarding forensically important information, e.g., possible causative events, agents and force directions. However the inconsistencies regarding the diagnosis of especially fractured eye lofts (contre-coup) was problematic and in the cases in which a differentiation between a fall on the back of the head or a blow is necessary the autopsy still seems to be the primary choice. Although the usage of different reconstructions improved the fracture visualisation, the agreement between the autopsy results and the CT images should be improved if CT scans of fractured neurocraniums are to be used for future retrograde biomechanical modelling and in order to be able to give a biomechanical approximation of the injury mechanism and involved forces.

## Abbreviations

CT: Computed Tomography; MPR: Multiplanar Reconstruction; MIP: Maximum Intensity Projection; MSCT: Multislice Computed Tomography.

## Competing interests

The authors declare that they have no competing interests.

## Authors' contributions

CJ: conception and design of the study, data acquisition, image and data analysis, draft of manuscript. BHB: image and data analysis. NL: conception and design of the study, image and data analysis, draft of manuscript. All authors read and approved the final manuscript.

## Pre-publication history

The pre-publication history for this paper can be accessed here:

http://www.biomedcentral.com/1471-2342/9/18/prepub
